# Knowledge graph of alpine skiing events: A focus on meteorological conditions

**DOI:** 10.1371/journal.pone.0274164

**Published:** 2022-09-21

**Authors:** Wei Tang, Xueying Zhang, Deen Feng, Yipeng Wang, Peng Ye, Hanhua Qu

**Affiliations:** 1 Public Meteorological Service Center, China Meteorological Administration, Beijing, China; 2 Key Laboratory of Virtual Geographic Environment, Ministry of Education, Nanjing Normal University, Nanjing, China; 3 Jiangsu Center for Collaborative Innovation in Geographical Information Resource Development and Application, Nanjing, China; 4 Urban Planning and Development Institute, Yangzhou University, Yangzhou, China; 5 College of Architectural Science and Engineering, Yangzhou University, Yangzhou, China; University of Innsbruck: Universitat Innsbruck, AUSTRIA

## Abstract

Alpine skiing, as an outdoor winter sport, is particularly vulnerable to the variation of meteorological conditions. Scattered and multi-source big data cannot be fully utilized to conduct effective decision analyses by conventional data analysis methods. Presently, knowledge graphs are the most advanced organization form of knowledge base, which can make explicit the complex relationships among different objects. Thus, introducing knowledge graph to the event management of alpine skiing is significant to improve the ability of risk prediction and decision-making. In this research, we analyze the components and dynamic characteristics of alpine skiing, and construct an “Object-Characteristic-Relation” representation model to express multi-level knowledge. Moreover, we propose a “Characteristic-value- Relationship” representation method based on the multi-source data, to construct the knowledge graph of alpine skiing. With the proposed method, comprehensive relationships between meteorological conditions and alpine skiing can be represented clearly, and support further knowledge reasoning for the event management under meteorological conditions. We have tested the utility of the proposed method in a case study of 2018 Winter Olympics in PyeongChang. The case study realizes an semi-automatic construction of knowledge graph for alpine skiing, provides decision supports for event risk managements, according to different meteorological conditions, and grounds a foundation for future knowledge graph construction of other large-scale sport events.

## Introduction

Alpine skiing event is a winter competitive sport that is extremely challenging be-cause of its high speed and high altitude. As an equipped outdoor sport, alpine skiing is sensitive to the real-time weather, and has extremely strict requirements to meteorological conditions [1–3]. For all eleven alpine skiing events in the 2018 Winter Olympics in PyeongChang, six events were postponed due to the adverse weather. The impacts of meteorological conditions on alpine skiing can be mainly concluded in two aspects: (1) Complex underlying surface in the alpine region leads to changeable weather. The unfavorable weather includes extreme low temperature, strong wind, sandstorm, haze, icing, or freezing rain, and may cause injuries to the related people [4]. (2) To maintain the competition environment, both natural snowfall and artificial snow are needed for alpine skiing. However, changing meteorological conditions may damage the ski trail, and make it unsatisfied to the basic requirements [5, 6].

People have already accumulated a large number of alpine skiing data, including the sport events data and natural environment data. However, a large number of objective laws are hardly found in the dynamic big data. In fact, risk prediction and event management are premises for the normal proceeding of alpine skiing events, which cannot be realized without the support of different types of knowledge. If the scattered and fragmented data resources can be integrated to form an interrelated and ordered structured knowledge system. The knowledge could be applied to the risk assessment and management of alpine skiing events. As a large semantic network, Knowledge Graph (KG), can be used to describe concepts, entities and their relationships in the objective world [7]. It simulates the thinking mode of human being, and uses a complex graph as the universal language to express objects and relationships [8]. The KG of Alpine Skiing events can systematically integrate related knowledge to reveal specific rules of alpine skiing events. Therefore, it has great potential to be applied in schedule arrangement, personnel scheduling, security rescue and event management. In this paper, the KG of Alpine skiing events is constructed to study the constraint mechanisms of meteorological conditions on alpine skiing events. Based on the analysis of the components of alpine skiing events, a knowledge representation model is constructed to describe the knowledge system. Furthermore, the mechanisms between meteorological conditions and alpine skiing events are integrated into the knowledge graph, which is essential to realize the inference of event management under specific meteorological conditions. The innovation of this study can be summarized as follows:

(1) The components of alpine skiing events are summarized according to the human cognitive habits. On this basis, we construct an “Object-Characteristic-Relation” representation model of alpine skiing events to express multi-level knowledge. Such framework can promote the transformation from multi-source data to knowledge.

(2) The “Characteristic value-Relation” representation method is proposed, which breaks through the limitation of conventional KG on describing complex relationships among different nodes. In addition, the multi-dimensional reasoning rules between meteorological conditions and alpine skiing events are embedded in the proposed KG, which is important to realize the risk prediction and emergency response in future applications.

The following sections are expanded as follows: Section 2 (related work) analyzes the research progress; Section 3 (construction process of knowledge graph) proposes the construction method for the KG of Alpine Skiing events; Section 4 (case study) takes the 2018 Winter Olympics in PyeongChang as a case study, and discusses the practical value of the proposed method; Section 5 (conclusions) presents the conclusion and future work.

## Related work

### The influence of meteorological conditions on alpine skiing

Outdoor sports are closely related to meteorological conditions (e.g. temperature, precipitation, wind power, haze, etc.). As one of the most sensitive factors, they may not only change the natural environment, but also affect the psychological or physiological state of athletes. Thus, larger sport events have stronger demands for accurate, effective and timely meteorological safeguard services [9, 10]. Significantly, the sensitivity of apparatus sports to meteorological conditions is much greater in winter than in summer, more stringent requirements are needed for outdoor winter sport events [11]. Therefore, both snow protection, running protection, event protection, and personal safety, have put forward severe challenges to the meteorological safeguards [12].

As a typical outdoor winter sport, holding a successful alpine skiing event also needs the support of good meteorological conditions. Among various meteorological conditions, snowfall and snow cover are two basic factors for alpine skiing. On the one hand, excessive snowfall makes athletes hard to control the descent speed. Compared with 2010 Vancouver Winter Paralympic Games, about one-third of athletes were injured, and the unfinished rates were also higher in 2014 Sochi Winter Paralympic Games. After analyzing the meteorological factors between these events, heavy snow and high humidity of ski resorts in Sochi are regarded as two major reasons [13]. On the other hand, if the snowfall is insufficient, alpine skiing events may have a large possibility to be cancelled. Indeed, such situation is not only suitable for large-scale events, but also affects average skiers. Generally, the skiing resorts can only attract publics by reducing the ticket price under bad weather [14, 15]. After a long-term study, the meteorological influencing factors on alpine skiing mainly includes precipitation, snowfall, visibility, temperature, relative humidity, wind speed, wind direction, etc. For instance, when the temperature is higher than 2°C or lower than -25°C, or showers with a strong wind faster than 15 m/s, the alpine skiing events may be suspended.

### Knowledge graph and its application

Knowledge graph is a knowledge base using graphs to represent comprehensive entities and relationships in the real world. KG commonly uses Resource Description Framework (RDF) to express knowledge, with a uniform triple as <head node, edge, tail node> [16]. Moreover, KG can be divided into two categories as General-purpose KG and Domain-specific KG due to different application fields. (1) General-purpose KG focuses on a large extent of knowledge and emphasizes more integration of general concepts and entities. At present, several representative general-purpose KGs are widely used including YAGO [17], DBpedia [18], Freebase [19], NELL [20], and Wikidata [21]; (2) As an important branch, domain-specific KG has higher requirements on the depth and accuracy of knowledge in a specific domain, and can provide targeted decision supports to solve problems [22]. However, more knowledge demands higher precision in information extraction and knowledge fusion, and increases the difficulty of knowledge reasoning.

In general, the KG can be constructed with the top-down or bottom-up approaches. To integrate more entities, a General-purpose KG is often constructed by the bottom-up approach [23]; while the construction of a Domain-specific KG combines both methods for a more complete conceptual knowledge model [24, 25]. There are several steps to construct a KG, including knowledge modeling, knowledge acquisition and knowledge application. Knowledge modeling is the logical basis of the entire process, which defines explicit formal specifications for the types and attributes of different concepts, entities and relationships [26]. Knowledge acquisition first extracts the information from a large number of structured, semi-structured or unstructured data, and then enhances the logic and representation of knowledge base through a series of post-processing procedures, such as entity disambiguation, entity integration, etc. [27, 28]. Knowledge application utilizes existing knowledge in the constructed KG for further knowledge reasoning and quality evaluation, which can be realized by the description logic reasoning, rule-based reasoning and case-based reasoning, etc. [29]. KG has widely use in many fields such as intelligent question answering, semantic search, intelligent recommendation, and decision analysis systems [30–32].

In recent years, domain-specific KG has been applied to medical, education, finance, e-commerce and other industries successfully. Jiang et al. proposed an analytical method to visualize the epidemic situation of COVID-19 interactively by using the geographic KG, which can analyze the dynamic patient-patient relationship, monitor real-time epidemic situation, and prevent high-risk groups [33]. Qi et al. constructed a meteorological and agricultural KG for the automatic generation of crop meteorological reports [34]. Liu et al. constructed a KG for typhoon disaster by Neo4j graph database, which can be used to reveal the distribution rules of typhoon [35]. Currently, only a few researches are conducted to build KGs in the domain of sport events. Several institutions have jointly developed the “Beijing Winter Olympics KG Resources and Q&A System” to provide real-time and convenient Q&A services for the Winter Olympics [36]. Quang Nguyen et al. constructed a recommendation system for sport events with natural language processing and unsupervised learning methods. To realize this function, the ontological framework and user characteristics are constructed by collecting data from online sports sites [37]. Rule-based systems have been extensively used in several applications and domains. In the domain of knowledge graph, there is a combination of ontology and rules. SWRL is a semantic web rule language that combines OWL ontologies with Horn Logic rules of the RuleML family of rule languages. However, key inference problems for SWRL are undecidable [38]. SPIN can represent SPARQL rules and constraints on Semantic Web models [39]. SHACL is a W3C-proposed language for expressing structural constraints on RDF graphs [40]. Although a portion of rule representations based on graph patterns have been proposed, most of them are based on the RDF language. For data structures with attribute graph structures, conceptual models capable of representing complex rules are needed.

In summary, KG has been applied to risk identification, sports knowledge Q&A and association analysis. But the KG of sport events is still in a preliminary stage, which is inclined to the storage and visualization of existing knowledge resources. Therefore, an important research topic in the field of sports events is how to effectively store, organize and manage large amounts of knowledge, and effectively leverage existing knowledge for exploitation. It allows KG to help with event management by correlating large amounts of weather data with event information.

## Construction process of knowledge graph

### Overall technical process

**Fig 1:**
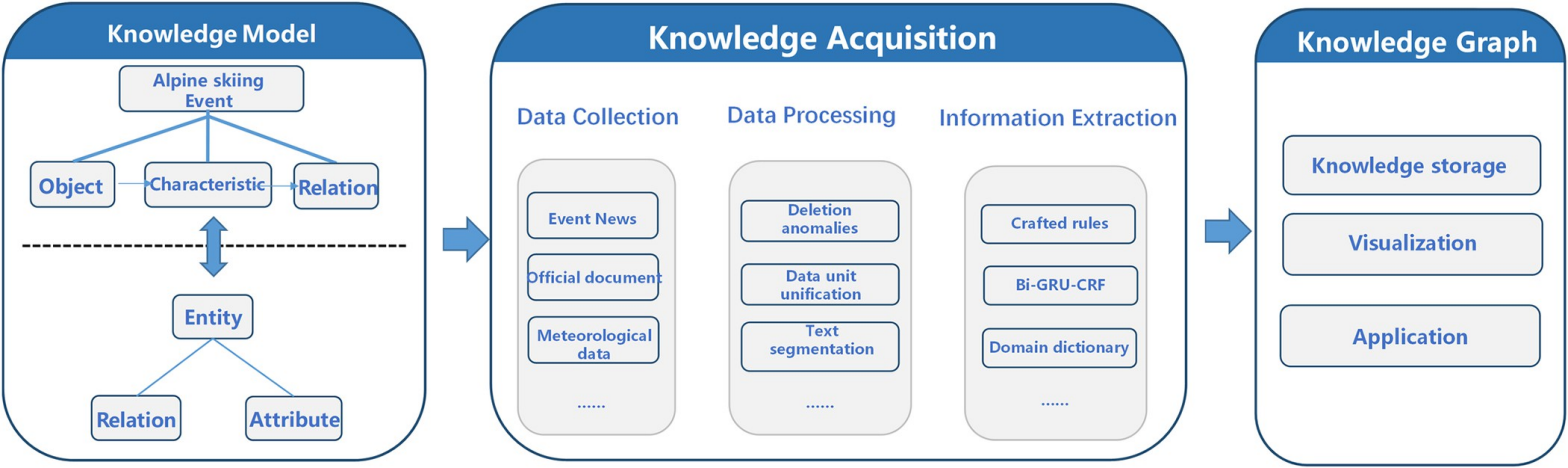
Flow chart for the construction of KG.

The KG of alpine skiing events adopts the top-down construction method, including three basic steps: construction of knowledge representation model, knowledge acquisition, and knowledge storage and management (Fig 1). We first analyze the components and dynamic characteristics of alpine skiing events, and consider the significant influence of meteorological conditions on alpine skiing events. On this basis, a multi-level knowledge representation model is constructed, named as “Object-Characteristic-Relation” model. Additionally, various relevant data (e.g. event management data, social activities and meteorological conditions, etc.) are collected to extract the entities and characteristics of alpine skiing events. At the meanwhile, the “Characteristic value-Relation” representation method is proposed to represent the multi-dimensional relationships among different objects. Furthermore, the graph database is used to organize and manage the fused entities and relationships, and the KG of alpine skiing events is constructed.

### Knowledge representation model

**Fig 2:**
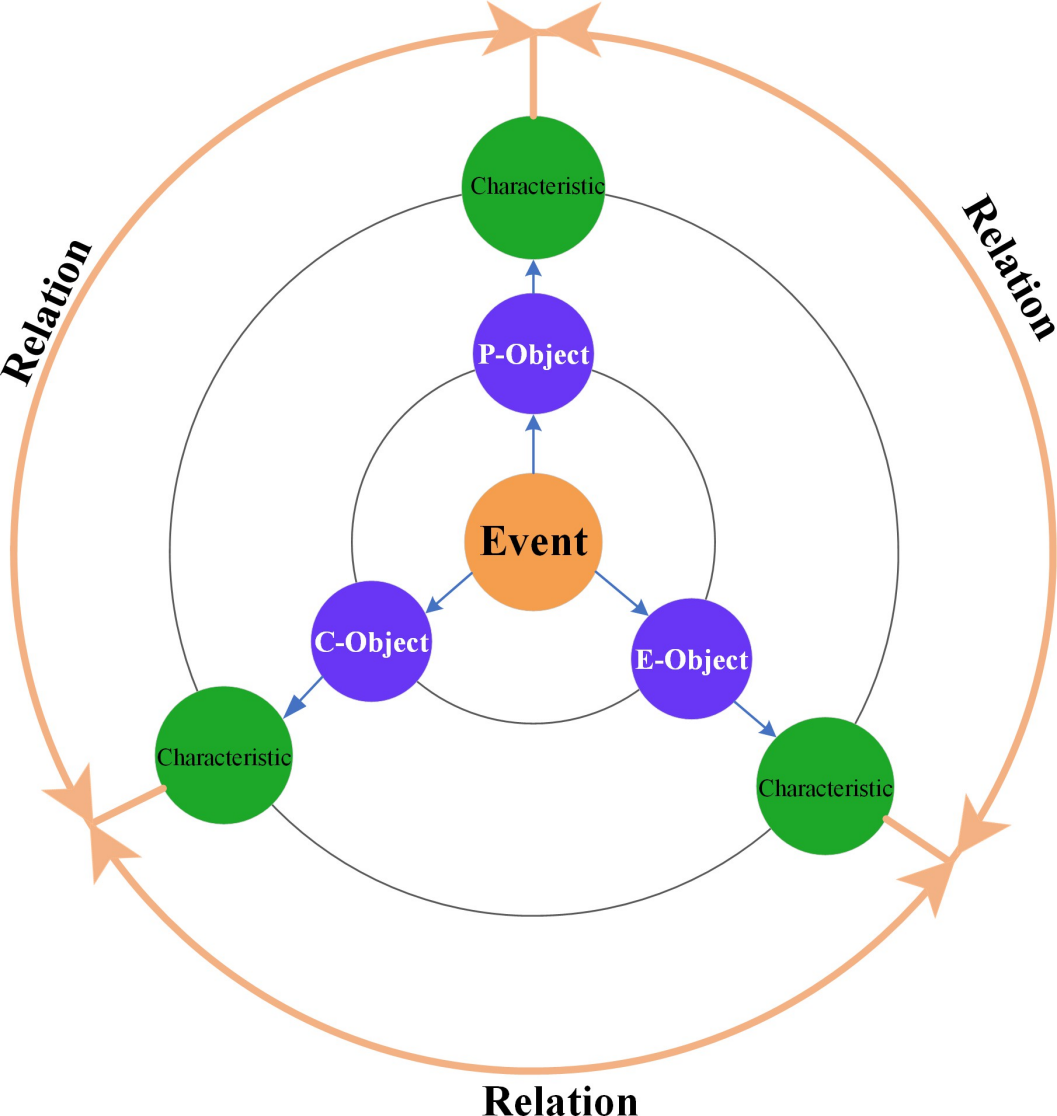
Knowledge representation model of alpine skiing events.

The knowledge representation model of alpine skiing events is a formal and structural abstraction of related knowledge. It represents time, place, people, and thing in a multi-level perspective, and emphasizes relationships between meteorological conditions and alpine skiing events. Three levels are defined in this model, which includes Object, Characteristic and Relation (Fig 2). With the proposed model, different types of objects can be highlighted, and the mutual relationships among various objects are described.

### 1. Object-level

**Fig 3:**
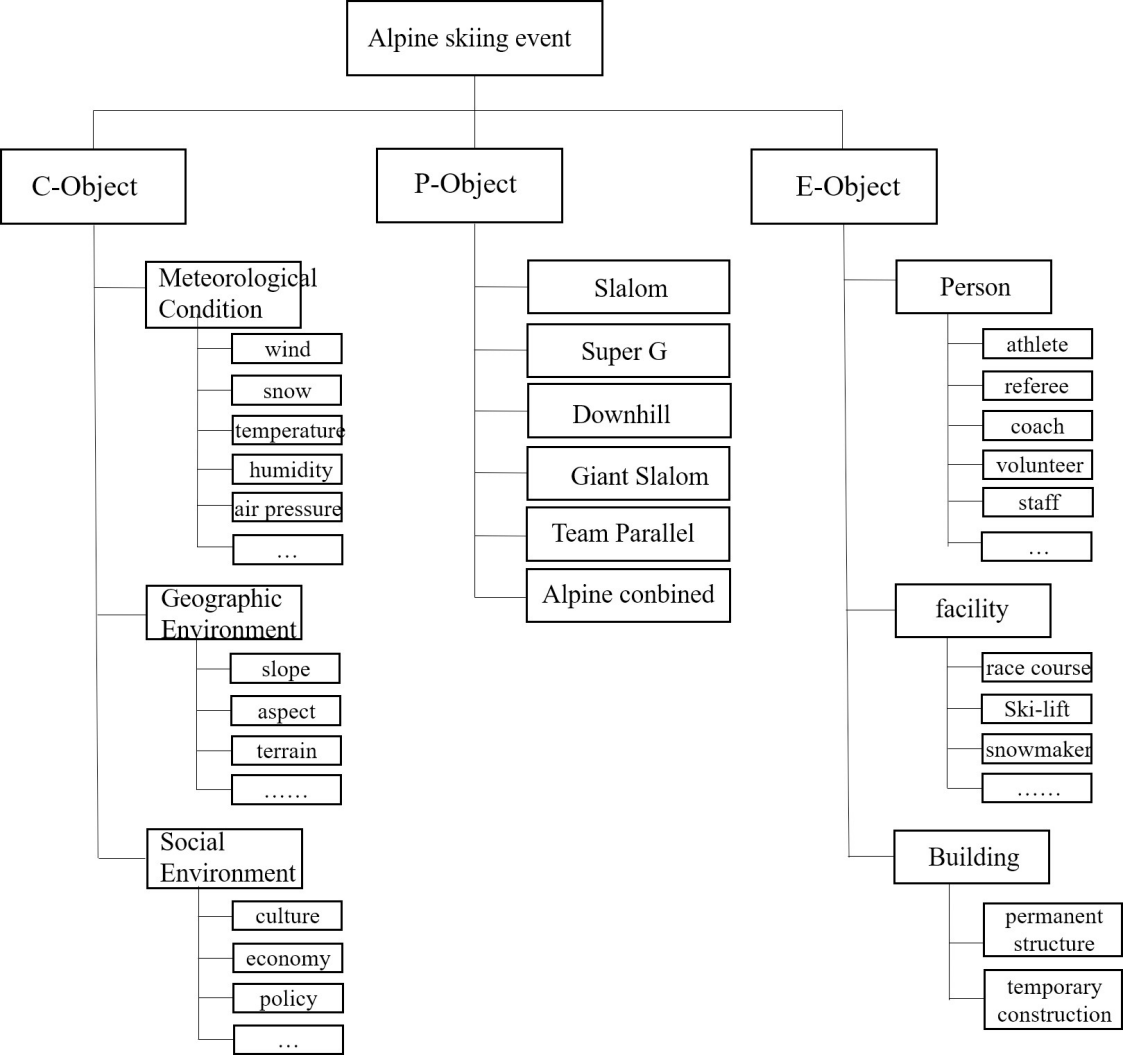
Object classification of alpine skiing events.

The Object-level includes various objects, such as competition events, competition venues, snow-making and snow-preserving equipment, transportation facilities, people (athletes, referees, other staffs) and natural or social environmental factors. Each object has different states under different spatiotemporal conditions, while each state is an existence of the object in a particular time and place representing the corresponding attributes and actions. The constituent objects of an alpine skiing event are complicated. According to the relationships between different objects, we divide objects into three kinds, namely primary object (P-Object), conditional object(C-Object), and effect object (E-Object) (Fig 3).

P-Object: Competitions are the most critical and dominant part of the alpine skiing events, named as P-Object. It has direct or indirect effects on C-Object and E-Object, and can be divided into six categories: Downhill, Slalom, Giant Slalom, Super G, Alpine combined, and Team Parallel.

C-Object: C-object involves natural and social environmental factors, which are the preconditions of alpine skiing events. As the important influencing factor on P-Object and E-Object, it includes meteorological condition, geographical environment and social environment. Among them, this paper mainly focuses on the meteorological conditions, which can be further divided into wind, rain, snow, temperature, humidity, visibility, etc.

E-Object: E-objects refer to the objects affected by P-Object and C-Object, including people, facilities, buildings, etc. People consist of athletes, referees, coaches, media staff, security staff, etc. Facilities include race tracks, snow-lifts, snow-making facilities, etc. Buildings include temporary and permanent buildings, such as temporary tents.

### 2. Characteristic-level

Each object includes four types of characteristics: time, space, attribute, and action. Time is used to represent the temporal information of each object, such as the starting and ending time of an event, the duration of wind and snowfall, etc. Space indicates the spatial information (location and geometry) of objects, including the location of an event, the referee’s work location, etc. Attributes are used to record the object’s properties (Table 1). Action is used to describe various activities occurred in this event. For example, the competition has normal progress, suspension, postponed (Table 2). The above four characteristics can describe the state of objects, and essential components of different interactive objects.

**Table 1:** Attribute of C-objects, P-objects and E-objects.

Object type	Object		Attribute
P-Object	Downhill		start time, competition venue, number of participants, etc.
	Slalom		
	Giant Slalom		
	Super G		
	Alpine combined		
C-Object	Meteorological conditions	wind	gust velocity, average wind speed, wind direction, etc.
		snow	snowfall, snow temperature, snow depth, etc.
		atmosphere	temperature, humidity, air pressure, etc.
		…	…
	Geographical Environment	terrain	altitude, slope
		location	coordinates, altitude
		climate	climate type
		…	…
	Social environment	culture	support degree of event.
		economy	financial situation.
		security	security conditions
		…	…
E-Object	Person	athlete	FIS code, name, rank, coach, competition item, etc.
		coach	coach name, contact, athlete, etc.
		volunteer	volunteer name, contact, service item, etc.
		referee	referee name, contact, service item, etc.
		staff	staff name, contact, tasks, etc.
		…	…
	Facility	Ski lift	lift name, transit time, carrying capacity, etc.
		race track	course name, start altitude, vertical drop, etc.
		power supply facilities	operating status, service scope, number, etc.
		water facility	
		…	…
	Building	permanent structure	building name, function, capacity, etc.
		temporary construction	

**Table 2:** Some actions of objects.

Object type	Object	Action
P-Object	Downhill	normal progress, postponed, cancelled, suspension, etc.
	Slalom	
	Giant Slalom	
	Super G	
	Alpine combined	
	Team Parallel	
C-Object	Meteorological conditions	light snow, heavy snow, high wind, heavy fog, low temperature, etc.
	Geographical Environment	landslides, debris flows, avalanches, etc.
E-Object	Person	work, rest, injury, etc.
	Facility	damaged, stopped using, operating.
	Building	Normal, damaged, destroyed.

### 3. Relation-level

Two types of relationships are proposed in the knowledge representation model of alpine skiing events. The first type of relation is relatively simple, structured as “Entity-Relation-Entity” or “Entity-Attribute-Attribute value”, such as “Men’s Slalom-Start Time-2018/2/22”, “Course-Course Name-Rainbow 1”. The second relation connects different objects with previous characteristics (time, space, attribute, and action). The mutual influences between objects are expressed by the relationships between different triples. Both attributes and actions can affect the advance of alpine skiing events. For instance, the attribute "Wind speed" can affect the action of Downhill, Slalom, and Giant Slalom. When the wind speed is faster than 17 m/s, the Downhill event should be cancelled. When the wind speed is slower than 11 m/s, a “normal progress” action would be associated with the Downhill node. Moreover, the “pause” action for Downhill is associated with “rest” action of athletes, and the “normal progress” action of Slalom is associated with the “operation” action of ski lifts.

### Knowledge acquisition

#### Data source and preprocessing

The data source of alpine skiing events can be divided into meteorological data and sport event data. The meteorological data include measured data, historical data, and predicted data issued by the weather advisory. The sport event data can be obtained from the official websites, online news, and social media. Since the multi-source data include structured, semi-structured, and unstructured data, the data collection methods should be customized accordingly. Specifically, the structured data can be mapped directly to RDF files using the D2R (Database to RDF) tool. Semi-structured data needs to parse the structure of web page, then design a matching model according to the elements, and finally obtain data with the web crawler. Unstructured data mainly come from the text data, which can be automatically extracted by information extraction techniques (e.g. Named Entity Recognition, Attribute Recognition, Relationship Extraction, etc.). The acquired data should be preprocessed to unify data units, and remove abnormal data or pausing words. For instance, the units of wind speed include m/s, km/h, mi/h, knots, but we only adopt m/s as a unique unit in this research.

### Entity extraction

Based on the characteristics of meteorological data and alpine skiing data, this paper adopts a combination of domain dictionary and deep learning methods to extract entities. The terminologies of alpine skiing events are extracted with the manually constructed domain dictionary (Table 3). Such dictionary involves name, level, type of different competitions. General vocabularies such as time, location are extracted with the Bi-GRU-CRF model [41].

**Table 3:** Examples for the labels of a domain dictionary.

Label type	Label	Label content
Ski type	SSLX	Alpine skiing, Cross-Country Skiing, Ski Jumps, etc.
Type of event	BSXM	Slalom, Giant Slalom, Downhill, etc.
Impact results	RES	Normal, reschedule, cancel, etc.
Influence factors	YXYS	high wind, heavy snow, thick fog, etc.
Event level	SSJB	Olympic Games, Championships, World cup, etc.

### Relation extraction

The relationships in alpine skiing events can be divided into simple relationships and compound relationships. The simple relationship can be obtained by several extraction rules, which includes the parent-child relationship, and other basic relationships between different objects, and characteristics (Table 4). The relationships between objects and characteristics are depicted from four aspects, namely time, space, attribute, and action. For instance, the node has a “contain” relationship with the "Downhill" node. The extraction method can be described as follows: First, the text is segmented into different words and the pause words are deleted according to the specific dictionary. The parts of speech are then utilized to filter the sentence and determine if it contains some words we need. Finally, the attributes of each entity are extracted from the text according to the given syntax rules.

**Table 4:** Examples of simple relations.

Node	Relation	Node
Temperature	value	-25°C
Visibility	value	200m
competition item	contain	Downhill
competition item	contain	Super G
Downhill	characteristic	Start time
Downhill	characteristic	Competition venue
Start time	value	2018/02/15
Competition venue	value	Jeongseon Alpine Centre

**Fig 4:**
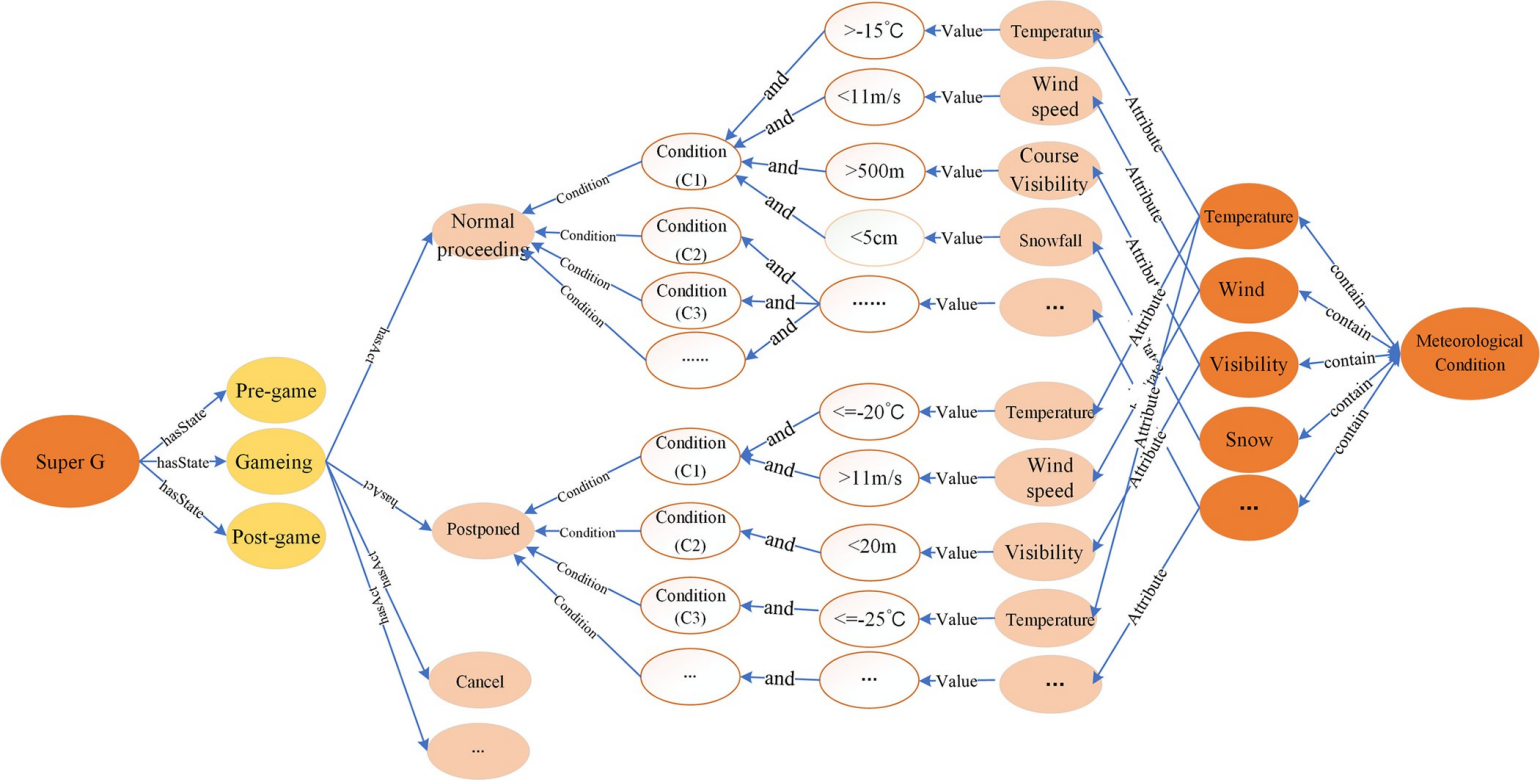
Diagram for the composite relation.

The composite relation is the relationship expressed by “condition” nodes in the KG of alpine skiing events, which is concretely showed as the interaction among P-Objects, C-Objects, and E-Objects (Fig 4). According to the proposed “Characteristic value-Relation” representation method, two conditional nodes are connected with characteristic values of different objects, and the influencing conditions between objects are represented through the combination of characteristic. Both rule extraction and artificial recognition methods are applied to obtain compound relations. The characteristics of each object are first extracted by the given rules, and then connected to the “condition” nodes manually. For alpine skiing events, same action conditions may be caused by more than one meteorological element. As shown in Fig 4, Super G contains “Normal proceeding”, “Postponed” and “Cancel” actions. Different actions can be triggered by different conditions. Taking meteorological conditions as an example, one of the conditions of “Normal proceeding”, c1, is consists of four meteorological factors, which are air temperature, wind speed, visibility of race tracks, and the amount of snowfall. For the conditions of “postponed”, they require the temperature low than -20°C and wind speed faster than 11m/s for c1; the visibility shorter than 20 m for c2, the temperature lower than -25°C for c3.

Knowledge fusion is an important step after the acquisition of entities and relationships, including entity alignment and entity linking. To realize entity alignment, the contextual similarity should be calculated and a professional dictionary needs to be built for alpine skiing and meteorology. Since there is a lack of corpus in alpine skiing, it is necessary to ensure the accuracy by combining artificial assistances. After completing the entity alignment, candidate targeted entities are selected from the knowledge base, and entities can be linked to correct target entities through similarity calculation and semantic matching. In addition, an artificial entity alignment can be used for a small-scale knowledge. For instance, the extracted race items, as well as their corresponding information are linked to P-Object and their relevant attributes or relationships.

### Knowledge storage

Rich alpine skiing data contain a large number of attributes and relationships. A graph database can represent these data from many dimensions, such as concept, attribute, and instance [42]. In the graph database, entities, attribute values are stored as nodes, and the sematic relationships are stored as edges [43]. Thus, structured knowledge triples can be mapped to the KG, and the KG can be further applied to specific domains.

**Fig 5:**
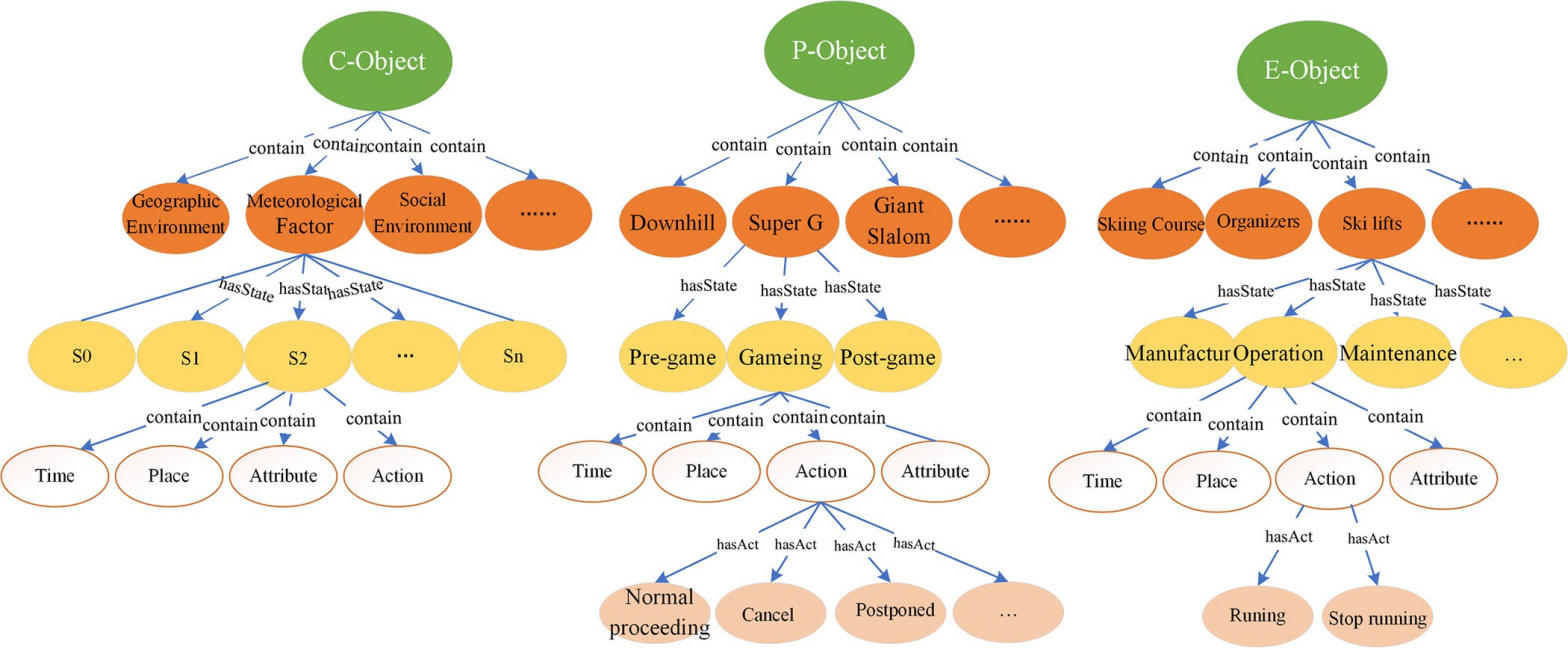
Schematic diagram of the KG of alpine skiing events.

Fig 5 shows a data storage in the KG of alpine skiing events. With this diagram, various nodes can be used to store objects, states, attributes, actions, etc. Different edges indicate the hierarchy and characteristics of different objects. Specifically, “P-Object” (green), is the abbreviation of primary-object, can be used to represent the main objects in this case. It contains various instances, such as Downhill, Giant Slalom, Slalom and other events (orange). For each instance, a “hasState” edge connects the instance with different state nodes (yellow). The state node includes several characteristic nodes, namely time, space, attribute and action. Taking “Gaming” state as an example, the action of this event includes Normal proceeding, Cancel, posted, etc. In addition, the representation methods for C-Object and E-Object are similar with the P-Object, using triples to express objects, states and various characteristics.

## Case study

### Study data

In this study, the alpine skiing events in the 2018 Winter Olympics in PyeongChang are selected as the study case. From February 8th to February 24th, 2018, the alpine skiing events in PyeongChang were held in two venues, Jeongseon Alpine Centre and Yongpyong Alpine Centre. 5 matches out of total 11 matches were rescheduled due to bad meteorological conditions since some facilities of the venues were damaged by extremely strong winds. Therefore, this event was chosen as a study case. Meteorological data and event achievements were collected to build the KG of alpine skiing events. Meteorological data is collected from the PyeongChang, including real-time hourly weather records and forecast data from the venues. The event achieves mainly includes the text from International Ski Federation website, the mainstream media websites, and the documents provided by the organizing committee.

According to the above-mentioned method of KG in section 3 (construction process of knowledge graph) Pre-processing was done at first to make the dataset standardized and accurate for the further processing. The C-Object, P-Object and E-Object are then extracted from the dataset. The entity name and attribute information was designed to directly extracted from structured dataset. After that, with the help of LAC tool and self-built domain dictionary, the text data are further processed. Words were segmented and their part of speech were tagged, the stop words were also removed. The object, characteristic, and relationship information are finally extracted and fused by relation template, and the triplet set of alpine skiing event knowledge is obtained. All the knowledge are stored and managed using the Neo4j database.

Authors should discuss the results and how they can be interpreted from the perspective of previous studies and of the working hypotheses. The findings and their implications should be discussed in the broadest context possible. Future research directions may also be highlighted.

### Analysis of result

**Fig 6:**
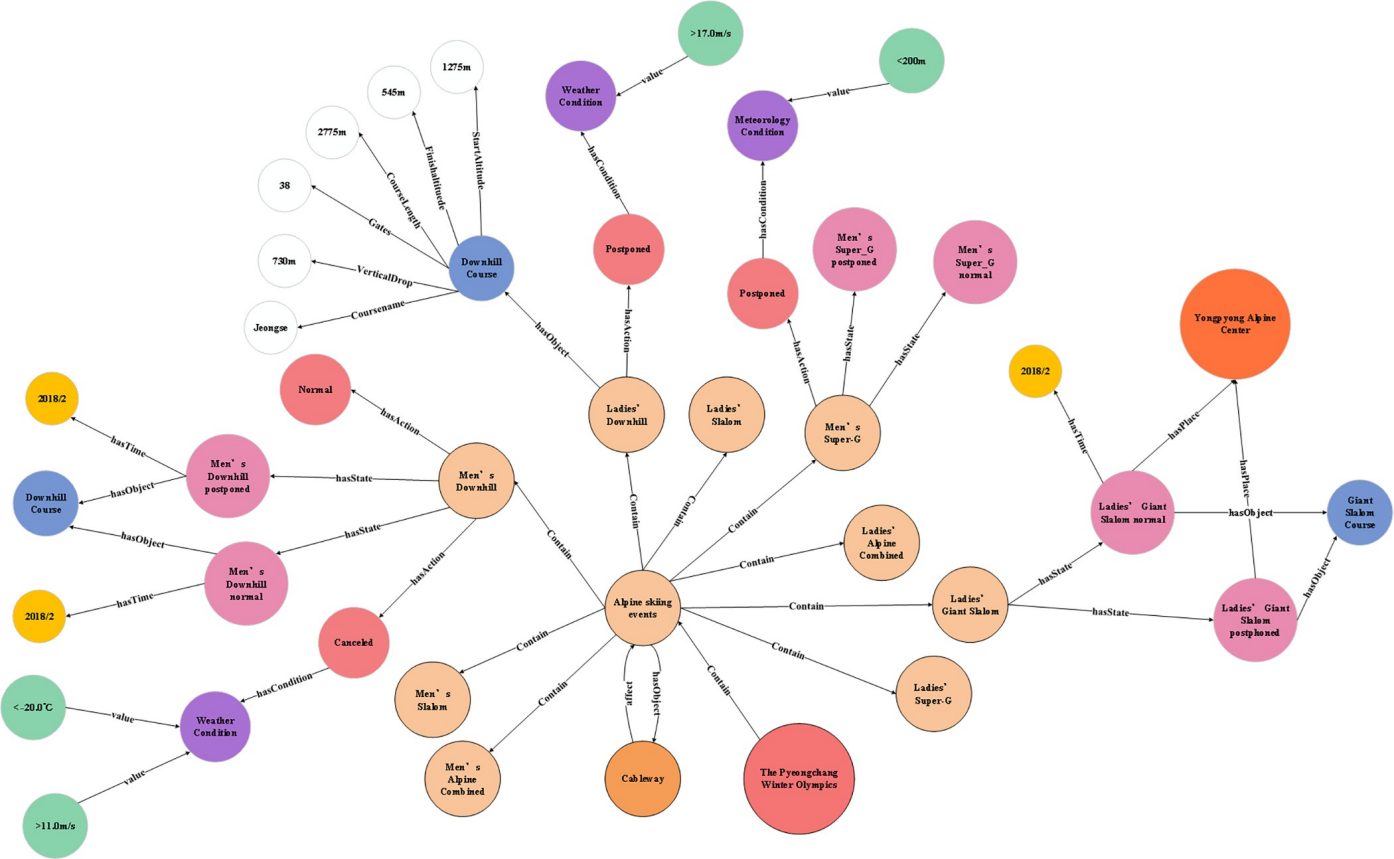
Part of the alpine skiing event KG (Neo4j visualization tool).

Neo4j graph database was used as the visualization tool to display the generated KG of alpine skiing event in the 2018 Winter Olympics in PyeongChang (Fig 6). In this KG, there are 11 kinds of P-Objects, 8 kinds of C-Objects, and 6 kinds of E-Objects. All objects have 175 characteristic nodes, 69 state nodes, and 180 relationships (Table 5). The experimental results show that P-Object has a relative high data integrity, which covers all events in this case. The C-Object covers major meteorological elements during the event, including temperature, wind speed, visibility, humidity, rainfall, and snowfall. It should be noted that some meteorological indexes are missing due to different data sources. E-Object mainly includes six types of objects, such as athletes, referees, competition venues, race tracks, cable cars, and snow-making equipment. The analysis on abnormal action of the subjects indicated that wind speed is the most important factor for the match. Downhill, Giant Slalom, Slalom, and Alpine combined events were rescheduled due to high wind speed, while the ski lift was halted during that time. We conducted random sampling statistics on the accuracy of different node and relationship extraction, and the results are shown in Table 6.

**Table 5:** Statistical of node types and relationship types of the constructed KG.

KG Elements	Numbers of Elements
P-Object type	11
C-Object type	8
E-Object type	6
Characteristic type	28
Relation type	23

**Table 6:** Accuracy of node and relationship extraction (random sampling).

ID	Data	Number of data	Number of errors	Accuracy
1	P-Object nodes	20	0	100%
2	C-Object nodes	50	6	88.00%
3	E-Object nodes	50	11	78.0%
5	Characteristic nodes	200	31	84.50%
6	Relations	200	27	86.50%

The query of historical event information is one main function of KG. In this case study, two types of tests are designed: (1) query the events affected by meteorological conditions; (2) query the impact conditions of target events.

**Fig 7:**
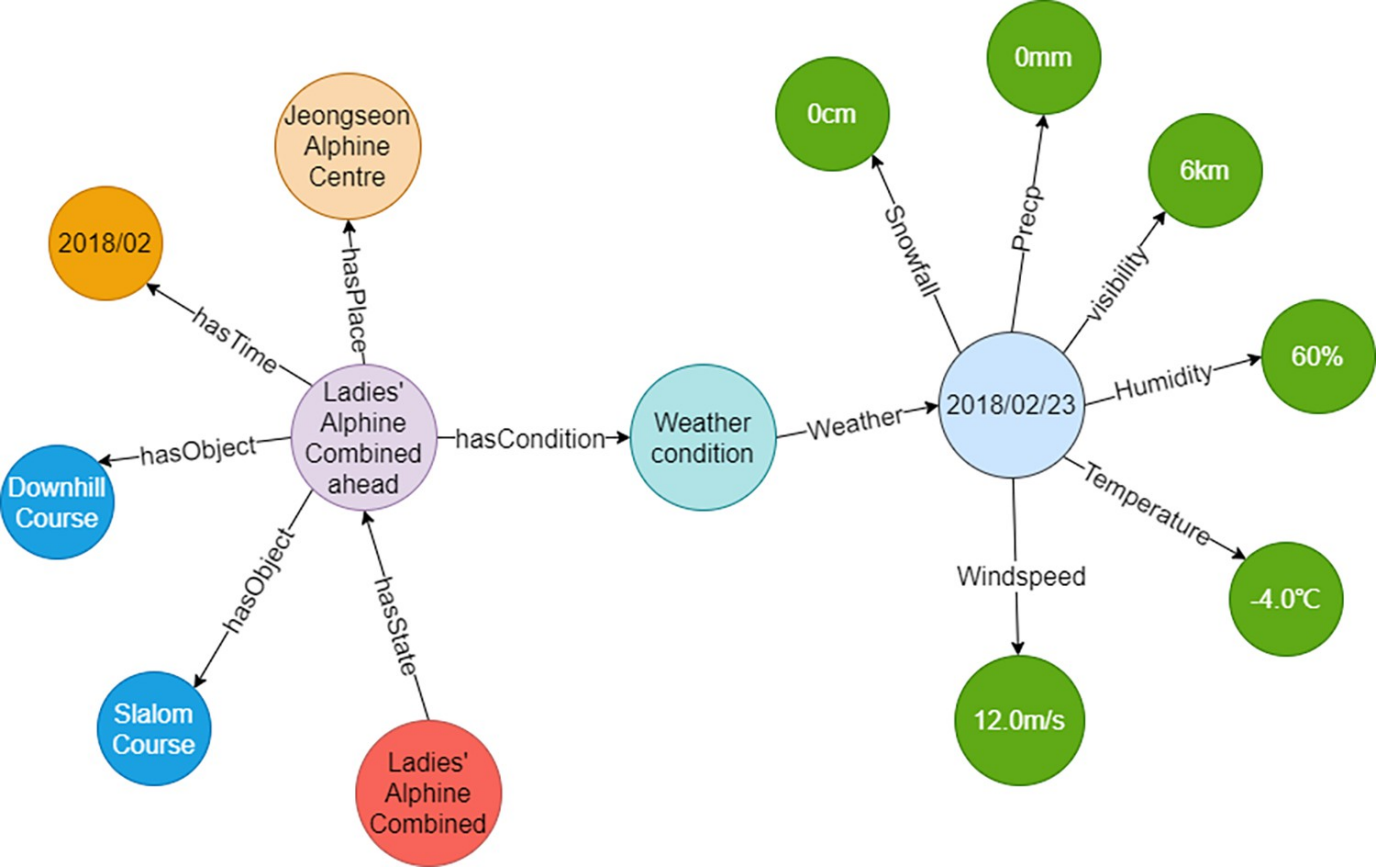
Schematic diagram of the query results—Ladies’ Alpine Combined be rescheduled.

For the first type of test, rescheduled events were found with different causes (e.g. wind speed, temperature, humidity, visibility, and snowfall). The results showed that Giant Slalom, Downhill, and Slalom competitions were postponed, and the Ski lifts in the resort were suspended due to the high wind speed. The Ladies’ Alpine Combined be rescheduled due to the bad weather (Fig 7).For the second type of test, multiple thresholds of five meteorological factors were retrieved to judge if the events can be hold normally. The results showed that when Super G, Giant Slalom, and Downhill events can be hold normally, the corresponding threshold of snowfall was 5cm, with the wind speed as 11m/s, and visibility as 500m. On the contrary, when the events were postponed, the threshold of snowfall was 30cm, with the wind speed as 17m/s, the visibility as 200m, and the temperature as -25°C.

The objects that will be affected according to the meteorological conditions can be also inferred by the KG. Three question types are designed in this experiment to verify the affected objects given: (1) When a single meteorological condition breaks through the threshold. (2) When multiple meteorological conditions break the threshold. (3) Non-quantitative meteorological conditions. The corresponding Cypher query modes in the experiment of these three types of questions are listed in Table 7.

**Table 7:** Different types of questions.

Question Types	Examples of questions	Cypher query template
Single meteorological element	Can the event be held when the wind speed is greater than 17m/s?	MATCH (a:wind),(b:event) WHERE a.speed = ’>17m/s’ AND b.name = ’Downhill’ (a)-[r:]->(b) RETURN a,b,r;
Multiple meteorological elements	Can the event be held if the wind speed less than grade 5, visibility more than 500 meters and snowfall less than 5cm?	MATCH (a:event),(b:wind),(c:visibility),(d:snowfall) WHERE b.speed = ’<grade 5’ AND c.visibility = ’>500m’AND a.snowfall = ’<5cm’ (a)-[r:]->(b) RETURN a,b,r;
Non-quantitative meteorological element	How will the equipment being affected when the wind speed is high?	MATCH (a:facility),(b:wind) = WHERE b.speed = ’high’ AND a.type = ’ski lifts’ (a)-[r:]->(b) RETURN a,b,r;

For the first question, wind speed, temperature, humidity, visibility, and snowfall were tested in Neo4j. The results show that the “Postponed” action node was returned when the wind speed was higher than 17m/s, and the same result was also returned to Downhill events. When the visibility is less than 200m for Super G events, a “Postponed” node was returned, and this node was related to visibility and Super G nodes (Fig 8). The results show that the competition cannot be carried out normally under such meteorological conditions, which is in line with the expected results. Moreover, the results of follow-up test on the amount of snowfall and temperature are also in accordance with common expectations.

**Fig 8:**
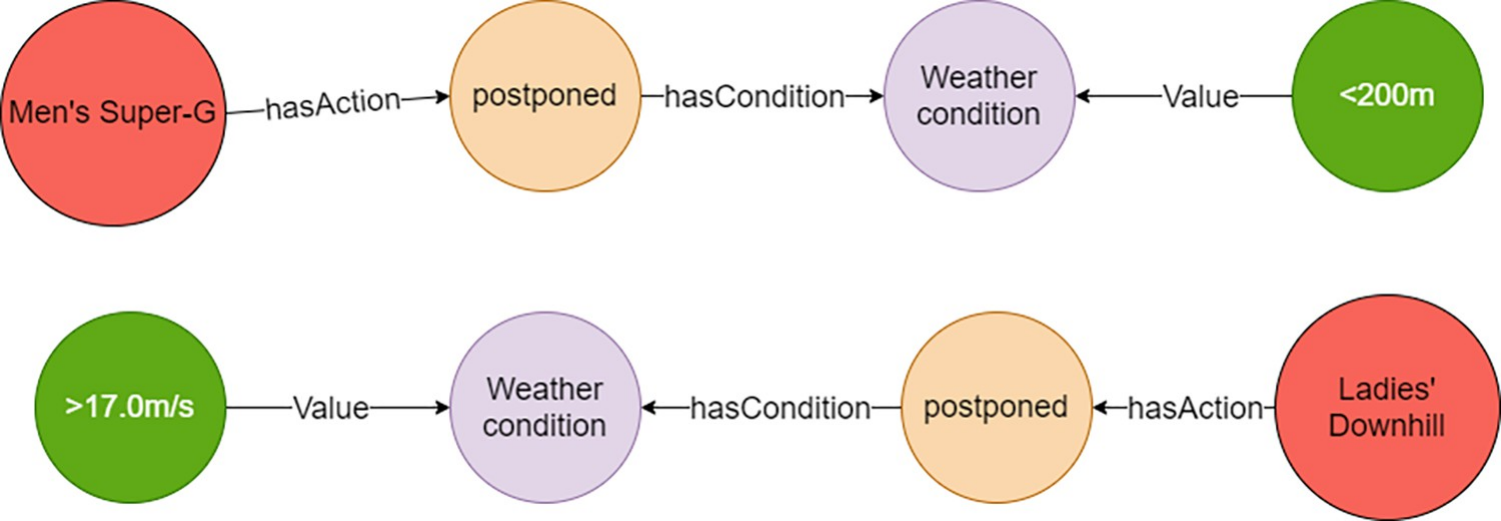
Single meteorological condition for event to be postponed.

**Fig 9:**
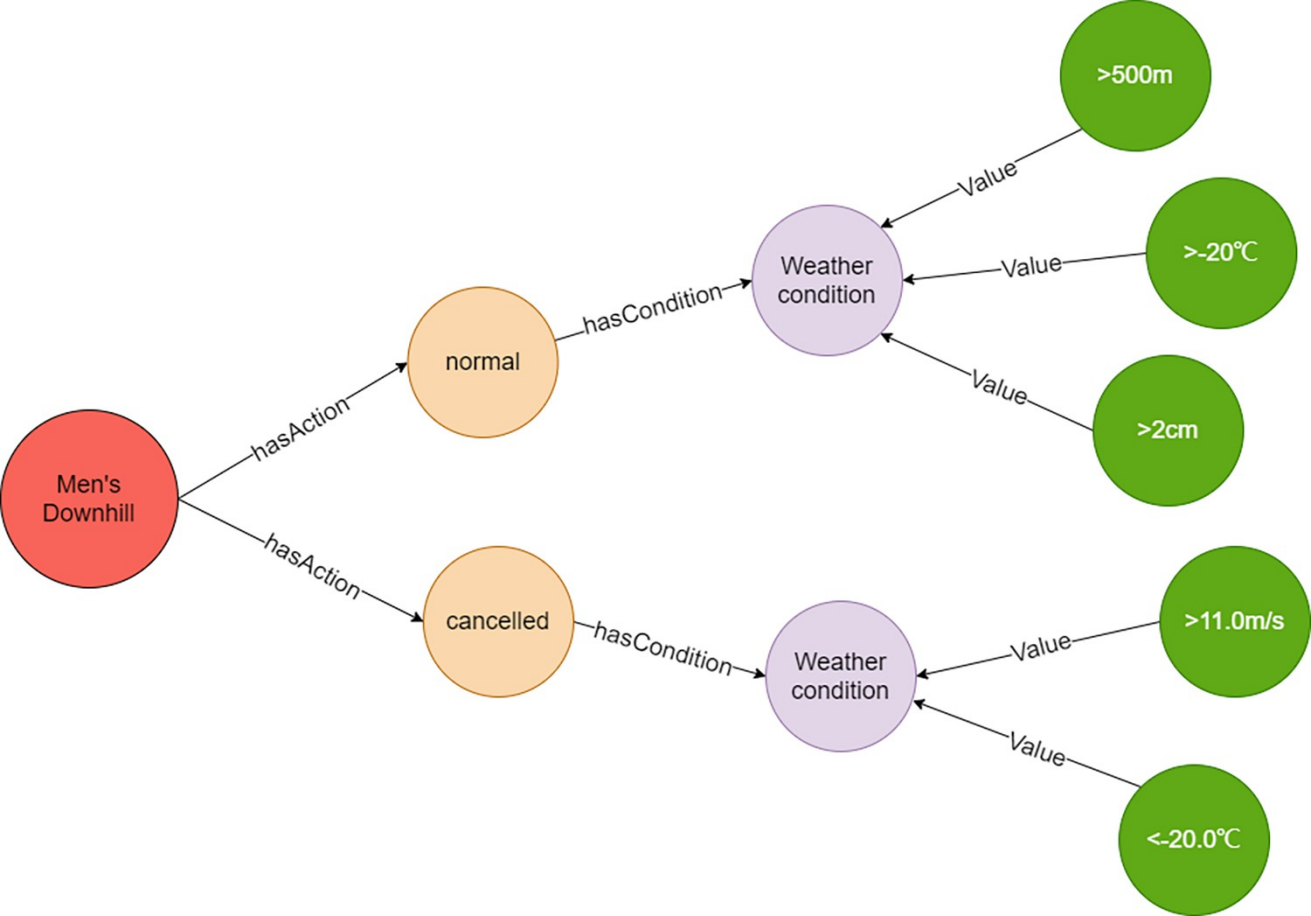
Multiple meteorological condition for event to be cancelled or normal.

To verify the second question, five meteorological elements (wind speed, temperature, humidity, visibility, and the amount of snowfall), were tested in Neo4j. In this case, Downhill events are used to verify the results (Fig 9). When the wind speed is faster than 11m/s and the temperature is lower than -20°C, a “Cancelled” action node is returned from the database. When the temperature is higher than -20°C, the visibility is larger than 500m, and the amount of snowfall is less than 2cm, the database returns a “Normal” action node. Based on the above results, the relationships between a combination of different meteorological conditions and the sport events can be searched in the database.

The third question is verified by searching non-quantitative meteorological conditions and their affected objects in the database, including low temperature, low visibility, heavy snow, strong wind, etc. The results show that we can use non-quantitative meteorological conditions to find the affected events, people, and facilities, while many related objects can be easily obtained by a single retrieval. Meanwhile, we selected four sets of data as listed in Table 8 as test cases and found two sets of errors. Therefore, the accuracy of weather risk assessment in rule-based knowledge graphs still needs to be improved.

**Table 8:** Results of meteorological risk assessment for 4 alpine skiing events.

Event ID	Risk Event	Meteorological conditions	Actual	Rule-based
				results
1	2018 Pyeongchang-Alpine Men-Downhill	Wind: 12.0 m/s	postponed	postponed
2	2018 Pyeongchang-Alpine Women-Slalom	Wind: 11.0 m/s	postponed	postponed
3	2018 Pyeongchang-Alpine Men-Super-G	Wind: 13.0 m/s	postponed	normal
4	2018 Pyeongchang-Alpine Women-Alpine-Combined	Wind: 10.0 m/s	postponed	normal

## Discussion

Alpine skiing events are composed of many objects such as skiing events, natural and social environment. The knowledge representation model of alpine skiing events is the fundament of constructing the KG of alpine skiing events. Previous researches on event models cannot be used directly to the domain of alpine skiing events. Based on the analyses of the components and dynamic characteristics of alpine skiing events, a knowledge representation model of alpine skiing events is constructed from three levels: object, characteristic, and relation. This model considers the multi-granularity characteristics of the related information in alpine skiing events, and unifies different characteristic information into each object. The proposed model can characterize different objects and their relationships in alpine skiing events, and express the attributes of objects and changing relationship through multiple state sequences.

Alpine skiing events are closely related to the meteorological conditions. On the one hand, alpine skiing events can only be held when a combination of meteorological conditions are met simultaneously. On the other hand, changing meteorological conditions may postpone or even cancel the event. Conditions are of great importance to facts. Existing conditional KGs have introduced condition triples, but ignore the latent semantic relations between fact and condition triples and the logical relationships among condition triples. The “Characteristic value-Relation” representation model proposed in this paper is suitable for representing the relationship between various meteorological conditions and alpine skiing events. Such representation method can reflect the relationships between different objects and display specific attributes and action of each relationship. In this way, multi-dimensional reasoning rules between meteorological conditions and alpine skiing events are embedded in the KG. Since it is operational and computable, the constructed KG can be used for risk prediction and the reasoning of emergency response in alpine skiing events.

The KG of alpine skiing events is not only a factual knowledge base to store existing events, but also a knowledge base for event risk prediction which incorporates reasoning rules of meteorological conditions. In alpine skiing events, organizers can use KG to obtain timely information, and identify potential risks that changing meteorological conditions may affect forthcoming events. Thus, KG has tremendous potential in security, rescue, and event or personnel scheduling of alpine skiing. For instance, when meteorological conditions reach specific values, the event organizing committee would give a proposal to cancel the competition. Besides, the proposed method in this research is a universal KG construction approach, which can be further extended to the management and application of other large-scale outdoor sport events such as ski jumping, cycling, sailing, etc.

## Conclusions

KG plays an increasingly important role in artificial intelligence. The KG of alpine skiing events can provide decision support for event management. Meteorological conditions are closely related to alpine skiing events and essential in constructing the KG. This paper focus on the meteorological conditions, and proposes a construction method for the KG of alpine skiing events. On the one hand, a multi-level knowledge representation model of alpine skiing events is constructed. The characteristics of different objects in the event are highlighted, and the evolution process of alpine skiing events is represented by the state sequence of several objects. On the other hand, multi-dimensional reasoning rules between meteorological conditions and alpine skiing events are embedded into KG by using the proposed “Characteristic value-Relation” knowledge representation model. This procedure achieves a reasoning process for the risk of holding alpine skiing events in specific meteorological conditions. The case study of alpine skiing events of the 2018 Winter Olympics in PyeongChang was carried out. The result can clearly and completely express the influence of meteorological conditions to the events, and can also describe the semantic relations among various elements. The case study reflects that KG can retrieve relationships between the meteorological conditions and the state of events, and make inference and prediction of potential influences of relevant factors. On this basis, it might support risk prediction and emergency decision-making of alpine skiing events under specific meteorological conditions in the future.

In the future research, the influence of geography society, and other environments on alpine skiing events can be further extended and integrated into the knowledge representation model. Furthermore, the application sceneries of KG should be extended in sports event management, such as intelligent Q&A, semantic retrieval, and automatic report generation.
